# Supplementary material for: Perspectives of stakeholders on barriers to COVID-19 protective behaviors adherence and vaccination among Myanmar migrant workers in southern Thailand: A qualitative study
Source: PLoS One. 2025 Mar 11;20(3):e0317714. doi: 10.1371/journal.pone.0317714 (PMC11896050; doi:10.1371/journal.pone.0317714)
Supplement: S1 Table — (DOCX) [file pone.0317714.s001.docx]

### **S1 Table.** Frequency of themes during interviews about barriers to adherence to COVID-19 protective measures

| **Group** | **Anonymized ID number** | **Theme 1: Complacency** | **Theme 2: Financial constraints** | **Theme 3: Health education and health promotion** | **Theme 4: Health service delivery** | **Theme 5: Lifestyle and habit-related barriers** | **Theme 6: Non-vaccine supply chain management** | **Theme 7: Language barrier** | **Theme 8: Non-compliance by migrant or employers** | **Theme 9: Fear** |
| --- | --- | --- | --- | --- | --- | --- | --- | --- | --- | --- |
| Myanmar migrant workers | Myanmar 01 | 0 | 0 | 0 | 0 | 3 | 0 | 0 | 0 | 0 |
|  | Myanmar 02 | 4 | 1 | 2 | 2 | 4 | 7 | 0 | 0 | 0 |
|  | Myanmar 03 | 2 | 0 | 2 | 0 | 7 | 1 | 0 | 0 | 0 |
|  | Myanmar 04 | 0 | 0 | 0 | 0 | 5 | 1 | 0 | 0 | 0 |
|  | Myanmar 05 | 0 | 0 | 5 | 2 | 9 | 0 | 0 | 0 | 0 |
|  | Myanmar 06 | 2 | 0 | 1 | 0 | 0 | 0 | 0 | 0 | 0 |
|  | Myanmar 07 | 0 | 0 | 0 | 0 | 6 | 0 | 0 | 0 | 0 |
| Thai employers | Employer 01 | 0 | 0 | 0 | 0 | 2 | 0 | 0 | 0 | 0 |
|  | Employer 02 | 0 | 0 | 0 | 0 | 0 | 0 | 2 | 0 | 0 |
|  | Employer 03 | 0 | 0 | 0 | 0 | 0 | 0 | 0 | 0 | 0 |
|  | Employer 04 | 0 | 0 | 0 | 0 | 0 | 0 | 0 | 0 | 0 |
|  | Employer 05 | 0 | 0 | 0 | 0 | 4 | 0 | 0 | 0 | 0 |
|  | Employer 06 | 0 | 0 | 0 | 0 | 0 | 0 | 0 | 0 | 0 |
| Thai  healthcare providers | Provider 01 | 0 | 0 | 0 | 0 | 0 | 0 | 0 | 0 | 0 |
|  | Provider 02 | 0 | 0 | 0 | 0 | 0 | 0 | 0 | 0 | 0 |
|  | Provider 03 | 0 | 0 | 0 | 0 | 0 | 0 | 0 | 0 | 0 |
|  | Provider 04 | 0 | 0 | 0 | 0 | 0 | 0 | 0 | 0 | 0 |
|  | Provider 05 | 0 | 0 | 0 | 0 | 0 | 1 | 1 | 1 | 1 |
|  | Provider 06 | 0 | 0 | 0 | 0 | 0 | 0 | 0 | 0 | 0 |
|  | Provider 07 | 0 | 0 | 0 | 0 | 0 | 0 | 0 | 0 | 0 |
|  | Provider 08 | 0 | 0 | 0 | 0 | 0 | 0 | 0 | 0 | 0 |
|  | Provider 09 | 0 | 0 | 0 | 0 | 0 | 0 | 0 | 0 | 0 |
