# Supplementary material for: Perspectives of stakeholders on barriers to COVID-19 protective behaviors adherence and vaccination among Myanmar migrant workers in southern Thailand: A qualitative study
Source: PLoS One. 2025 Mar 11;20(3):e0317714. doi: 10.1371/journal.pone.0317714 (PMC11896050; doi:10.1371/journal.pone.0317714)
Supplement: Attachment — Submitted filename: R1_Response Manuscript 3 Quali_202411209_Final.docx [file pone.0317714.s006.docx]

**Response to Reviewer Comments**

| **Comment** | **Response** |
| --- | --- |
| **Editor's comment** |  |
| EDITOR’S COMMENT  1. We note that the grant information you provided in the ‘Funding Information’ and ‘Financial Disclosure’ sections do not match. When you resubmit, please ensure that you provide the correct grant numbers for the awards you received for your study in the ‘Funding Information’ section. | RESPONSE  We thank the reviewer for the comment, and we have checked the grant numbers accordingly. |
| EDITOR’S COMMENT  2. We note that you have indicated that there are restrictions to data sharing for this study. PLOS only allows data to be available upon request if there are legal or ethical restrictions on sharing data publicly. For more information on unacceptable data access restrictions, please see http://journals.plos.org/plosone/s/data-availability#loc-unacceptable-data-access-restrictions.  Before we proceed with your manuscript, please address the following prompts:  a) If there are ethical or legal restrictions on sharing a de-identified data set, please explain them in detail (e.g., data contain potentially identifying or sensitive patient information, data are owned by a third-party organization, etc.) and who has imposed them (e.g., a Research Ethics Committee or Institutional Review Board, etc.). Please also provide contact information for a data access committee, ethics committee, or other institutional body to which data requests may be sent.  b) If there are no restrictions, please upload the minimal anonymized data set necessary to replicate your study findings to a stable, public repository and provide us with the relevant URLs, DOIs, or accession numbers. For a list of recommended repositories, please see  https://journals.plos.org/plosone/s/recommended-repositories. You also have the option of uploading the data as Supporting Information files, but we would recommend depositing data directly to a data repository if possible.  We will update your Data Availability statement on your behalf to reflect the information you provide. | RESPONSE  (a) We have checked the IRB protocols of this study and found no restrictions regarding sharing of qualitative in-depth interview data.  RESPONSE  (b) We will upload the final (analyzed) data set in excel to the submission system as a supplementary information file. |
| EDITOR’S COMMENT  3. We note that this data set consists of interview transcripts. Can you please confirm that all participants gave consent for the interview transcript to be published?  If they DID provide consent for these transcripts to be published, please also confirm that the transcripts do not contain any potentially identifying information (or let us know if the participants consented to having their personal details published and made publicly available). We consider the following details to be identifying information:  - Names, nicknames, and initials  - Age more specific than round numbers  - GPS coordinates, physical addresses, IP addresses, email addresses  - Information in small sample sizes (e.g. 40 students from X class in X year at X university)  - Specific dates (e.g. visit dates, interview dates)  - ID numbers    Or, if the participants DID NOT provide consent for these transcripts to be published:  - Provide a de-identified version of the data or excerpts of interview responses  - Provide information regarding how these transcripts can be accessed by researchers who meet the criteria for access to confidential data, including:  a) the grounds for restriction  b) the name of the ethics committee, Institutional Review Board, or third-party organization that is imposing sharing restrictions on the data  c) a non-author, institutional point of contact that is able to field data access queries, in the interest of maintaining long-term data accessibility.  d) Any relevant data set names, URLs, DOIs, etc. that an independent researcher would need in order to request your minimal data set.  For further information on sharing data that contains sensitive participant information, please see: https://journals.plos.org/plosone/s/data-availability#loc-human-research-participant-data-and-other-sensitive-data  If there are ethical, legal, or third-party restrictions upon your dataset, you must provide all of the following details (https://journals.plos.org/plosone/s/data-availability#loc-acceptable-data-access-restrictions):  1. A complete description of the dataset  2. The nature of the restrictions upon the data (ethical, legal, or owned by a third party) and the reasoning behind them  3. The full name of the body imposing the restrictions upon your dataset (ethics committee, institution, data access committee, etc)  4. If the data are owned by a third party, confirmation of whether the authors received any special privileges in accessing the data that other researchers would not have  5. Direct, non-author contact information (preferably email) for the body imposing the restrictions upon the data, to which data access requests can be sent | RESPONSE  We confirm that all participants gave verbal informed consent prior to data collection. We also confirm that the transcripts do not contain any potentially identifiable information (i.e., names and derivatives of names, specific age, specific date). We also redacted all circumstantial information that may allow for indirect identification of the participants or locations from the study findings. We did not collect ID numbers, GPS coordinates, physical addresses, IP addresses, or email addresses. |
| **Additional Editor Comments:** |  |
| EDITOR's COMMENT  4. Use the COREQ checklist for qualitative interviews and attach it as a supplementary document. | RESPONSE  We thank the editor for the suggestion, and we have uploaded the COREQ checklist for our study as a supplementary information document accordingly. |
| EDITOR's COMMENT  5. Introduction: It would be better to explain the COVID-19 waves and the epidemic situation in Thailand for clearer understanding to the readers. Was data collection conducted during the peak of the epidemic, or before or after a major wave? | RESPONSE  We thank the editor for the suggestion. We added about the COVID-19 waves and the epidemic situation in Thailand to the INTRODUCTION section as follows (Line 69-72):  “Thailand experienced five distinct waves of COVID-19 between 2020 and 2022, where the fifth wave of COVID-19 occurred in late 2022, and included the emergence of more transmissible variants like Alpha and Delta [10]. As of 13 April 2024, Thailand had a cumulative total of 4,770,149 confirmed cases with 34,586 deaths [11].” |
| EDITOR's COMMENT  6. Introduction: Information regarding the importance of adhering to COVID-19 protective behaviors and vaccination is missing. | RESPONSE  We thank the editor for the suggestion. We added about information regarding the importance of adhering to COVID-19 protective behaviors and vaccination to the INTRODUCTION section as follows (Line 79−83):  “Public health interventions to prevent and control COVID-19 have included hand hygiene, face covering use, social distancing in public places, isolation, travel restrictions, vaccination, etc. [22–25]. Different interventions affect disease transmission in different ways [26–28], and a comprehensive strategy is deemed needed in order to effectively control COVID-19 infection [29].” |
| EDITOR's COMMENT  7. Were the migrant workers you collected data from legal or undocumented workers? Legal and undocumented migrant workers may face different barriers. | RESPONSE  We allowed both types of migrants. But the migrants that we interviewed were all documented. We realize that such circumstances also limited the generalizability of our findings and have added the following remarks to the DISCUSSION section (in the “Strengths and Limitations” sub-section) as follows (Line 457-461):  “Firstly, although our protocols did not exclude undocumented migrants, we were only able to recruit documented migrants working in manual labor occupations common in southern Thailand. These circumstances limited the generalizability of our study findings. The barriers among undocumented migrants might have been vastly different from those reported by our participants.” |
| EDITOR's COMMENT  8. I am curious about the accuracy of Google Translate in translating Burmese and Thai texts into English. Did the study team review and correct the translations after using the tool? | RESPONSE  Yes. HH, a native speaker of Burmese, checked the accuracy of the translation and corrected the mistakes manually. WW, a native speaker of Thai, also performed similar procedures for the transcripts of interviews with the employers and healthcare workers.  We have added the following remarks to the METHODS section (in the "Data management and analysis" sub-section) as follows (Line 202-203):  “HH then manually checked the translations and corrected the parts deemed to be inaccurate.”  We have added the following remarks to the METHODS section (in the "Data management and analysis" sub-section) as follows (Line 207-208):  “A co-investigator and the corresponding author (WW) who spoke Thai as a native language, manually checked the translations and corrected the parts deemed to be inaccurate.” |
| EDITOR's COMMENT  9. Provide the background characteristics of the participants for all three categories. | RESPONSE  We have added the table of background characteristics of study participants in the RESULTS section in **Table 1**. (Page 13). |
| EDITOR's COMMENT  10. Currently, the investigators have analyzed the information based on the type of participant. Please consider analyzing the data by theme, covering all stakeholders. For example, under the theme of health education barrier, presenting perspectives from migrant workers, employers, and healthcare providers would make the findings easier to understand. | RESPONSE  We thank the reviewer for the comment. The questions were drafted separately for each stakeholder group and the probing differed by groups. Therefore, data may not be completely comparable between groups. |
| EDITOR's COMMENT  11. Move Tables 3 and 4 to the supplementary section. | RESPONSE  We thank the editor for the comment. We have changed Table 3 into Supplementary Table 1 and changed Table 4 into Supplementary Table 2. |
| **Reviewers' comments:** |  |
| **Reviewer #1:** |  |
| REVIEWER’s COMMENT  Thank you very much for your efforts in adding new information on perspectives regarding COVID-19 protective behaviors and vaccination among Myanmar migrant workers, Thai health care providers and Thai employers. In the attachment, I included my recommendations. | RESPONSE  We thank the reviewer for the comments, and we have attempted to address them on a point-by-point basis. |
| **Introduction** |  |
| REVIEWER’s COMMENT  1. Line 65-67: The statement mentions that 1.65 million migrants from Myanmar are in Thailand. It will be better to clarify the year form which this data was sourced. | RESPONSE  We thank the reviewer for the comment. We have updated the statistics of Myanmar migrant workers in the INTRODUCTION section as follows (Line 65-68):  “The majority of migrants in Thailand come from neighboring Myanmar with 1.65 million registered workers in 2021 according to the statistics from the Department of Employment, Ministry of Labour, Thailand, [8], most of whom work in low-skilled jobs such as construction, factories, and fisheries [9].” |
| REVIEWER’s COMMENT  2. Please consider adding information about the general health-seeking behaviors of migrants in the study area, especially focusing on irregular migrants. This could provide insights into their specific challenges in accessing COVID-19 preventative measures. | RESPONSE  We thank the reviewer for the comment. We have added the information about general health-seeking behaviors of migrants from previous studies in the study area in the INTRODUCTION section as follows (line 92-95):  “These challenges are further compounded by previous studies conducted in Southern Thailand, which highlighted unsatisfactory health-seeking behaviors among Myanmar seafarers in Pattani province [32], as well as factory, construction, and rubber trapping workers in Songkhla province [33].” |
| **Methods** |  |
| REVIEWER’s COMMENT  3. Line 98-99: It would be beneficial to include information on the non-pharmacological COVID-19 preventative interventions and restrictions in place during the study period, as these had evolved over time. | RESPONSE  We thank the reviewer for the comment. We have added information on non-pharmacological COVID-19 protective measures and restrictions in our study area in the METHODS section (“Study Design and Setting” sub-section) as follows (Line 115-120):  “During the COVID-19 pandemic (2020–2022), the industries in Southern Thailand were transiently affected, and the government issued several non-pharmacological measures, including social distancing, compulsory mask wearing, travel restrictions, and workplace disinfection, to prevent further spread among the workers. In late 2021, the government offered vaccinations to migrant workers free of charge as part of an inclusive vaccine policy [35]. ” |
| REVIEWER’s COMMENT  4. Line 99-101: It might be useful to explain why these cities were chosen for the study. It would be better to describe the number of migrant workers in those cities. I checked the cited reference, and it is in Thai language only. | RESPONSE  We thank the reviewer for the comment. We have added information about the rationale on choosing the study setting and the number of Myanmar migrant workers in that area according to the statistics from the Department of Employment, Ministry of Labour, Thailand, in the METHODS section (“Study Design and Setting” sub-section) as follows (Line 121-128):  “Study areas included the cities of Hat Yai city of Songkhla province and Pattani city of Pattani province in southern Thailand. Songkhla and Pattani provinces are major industrial commercial area of the Southern Thailand with many factories that process rubber, wood and seafood as well as numerous construction and fishery sites [36]. Many Myanmar migrant laborers live in these provinces, with different ethnic groups, religions, and cultures. According to the 2021 Thailand Employment Statistics, there were 19,810 and 4,122 legal Myanmar migrant workers granted by cabinet resolution in Songkhla and Pattani provinces, respectively [37].” |
| REVIEWER’s COMMENT  5. Line 116-118: It was mentioned that purposive sampling was used. Providing more detail on how key informants and participants were identified could be more informative. | RESPONSE  We thank the reviewer for the comment. We have added detail information on detail on how key informants and participants were identified in the METHODS section (“Study participants” sub-section) as follows (Line 145-153):  “Myanmar migrants were identified with the help of the key informants from the Migrant Workers Right Network (MWRN) and the Stella Maris Organization. These key informants were Myanmar Nationalities who had been working in the study area for more than 10 years. They could well acquaint with the Myanmar migrant population within Hat Yai and Pattani and could provide information on places of worksite and residence, types of occupations, legal status, approximate population sizes, and their lifestyles. For Thai employers and Thai healthcare providers, they were also identified by the Thai research coordinator and invited via official request letters, emails, or phone calls and invited to participate in individual in-depth interviews.” |
| REVIEWER’s COMMENT  6. It will be better to explain how cultural nuances were handled during translation and interviews. | RESPONSE  We thank the reviewer for comment. We assigned Thai reviewers to work only with Thai participants, and our Burmese interviewer (HH) only work with the Myanmar migrant participants. This enabled conversations to flow freely and in a manner that seems natural to the participants. This also enabled interviewers to probe for more information effectively. HH’s middle-class majority-Burmese background might have induced some hesitancy, social desirability, and response acquiescence in the initial period of conversation with migrant participants. However, HH made attempts to build rapport and noticed the changes in demeanors and responses during the course of the interview. HH did not notice any attempt at implicit communication in the interviews.  In our study, cultural nuances were managed during translation by careful translation which only focused on the language but also the cultural context of the participants. This ensured that the translations were not only accurate but also culturally relevant.  With regard to translation, we wish to first make a correction. HH’s translation of the Burmese transcript into English was done using ChatGPT instead of Google Translate, as HH compared the function of the two platforms and deemed ChatGPT to have a higher level of accuracy, including the ability to manage nuances. The Thai translation was done using only Google Translate based on the experience of the corresponding author (WW).    Therefore, we have included the following texts to the METHODS section (“Data Collection” sub-section) as follows (Line 179-184):  “We matched the nationality of the interviewers and participants in order to reduce potential language barriers, manage cultural nuances, and enable more effective probing. At the time of the study, HH was a male PhD student with a background as a public health physician. During the interview, HH made attempts to be conversational and build rapport with the participants in order to lower the potential social desirability and response acquiescence.”  We have also included the following texts to the METHODS section (“Data Management and Analysis” sub-section as follows (Line 199-202) :  “For in-depth interviews of the migrant workers in Burmese, the primary investigator (HH) who spoke Burmese as a native language transcribed the interview to Burmese texts, then used an artificial intelligence system (ChatGPT) to translate the Burmese texts into English.”  We have also included the following texts to the METHODS section (“Data Management and Analysis” sub-section as follows (Line 209-212):  “The decision to use ChatGPT to translate Burmese texts was made after HH made an assessment of both ChatGPT and Google Translate and found that ChatGPT had a higher accuracy and a better ability to manage cultural nuances. The decision to use Google Translate for the Thai texts was based on WW’s prior experiences with the validation of translated texts.” |
| REVIEWER’s COMMENT  7. Line 154-161: Using machine-assisted translation tools like Google Translate for initial translations is practical, but it might be worth discussing any limitations or potential inaccuracies associated with this approach or were there any steps taken to ensure the reliability of the translations. | RESPONSE  We thank the reviewer for the comment. We have added the following remarks to the DISCUSSION section (Line 464-469):  “Thirdly, in our study, the transcription and translation of the interviews were different for those of the Myanmar migrants and those of the Thai employers and healthcare workers. The choices were based on the subjective judgment of the investigators. Such practice might have introduced potential inaccuracies. However, considering that we used verbatim texts for our analyses, we anticipate these differences to be small, particularly in the context of the broader study findings.” |
| REVIEWER’s COMMENT  8. Line 166-172: It might be helpful to provide more detail on the criteria used for identification of themes, sub-themes, and codes.  It would be better to specify how VC’s (infectious diseases specialist) input was integrated into the final analysis and how any disagreements (if any) between VC and primary investigators were resolved. | RESPONSE:  We thank the reviewer for comment. We have added information on how themes, sub-themes, and codes were identified in the “METHODS” section (“Data management and analysis” sub-section) as follows (Line 218-219):  “The two investigators then separately identified codes, sub-themes, and themes, based on the frequency of occurrence and significance, relevance to research objectives, cultural and contextual sensitivity [44–46].”  Regarding VC’s input: We asked VC to look through our findings and asked for his comments, and VC simply said that he concurred with our findings and had nothing further to add. Thus, to clarify this point, we have added the following remarks to the “METHODS” section (“Data management and analysis” sub-section) as follows (Line 224-226):  “VC informed the investigators that he concurred with the interpretation and the conclusion and that he had no further comments. The investigators then made coding trees to summarize the findings.” |
| **Result** |  |
| REVIEWER’s COMMENT  9. Line 185-188: It would be helpful to include any relevant socio-demographic characteristics that might influence the findings or interpretations of the results. | RESPONSE  We thank the reviewer for the comment. We have added the table of background characteristics of study participants in the RESULTS section in **Table 1**. (Page 13). |
| **Discussion** |  |
| REVIEWER’s COMMENT  10. Line 270-283: It would be beneficial to explore the fear of vaccination in more depth. How does this fear differ across different socioeconomic groups within the migrant worker population? Adding these details would provide more context for your findings and potentially point toward targeted interventions for specific subgroups. | RESPONSE:  We thank the reviewer for the comment. We have discussed more about fears of vaccination and its financial implications, along with possible interventions in the DISCUSSION section as follows (Line 400-406):  “Our study only included migrants with relatively low socioeconomic status. Studies have found that context of migration [67], language barrier and employer support [57] are associated with COVID-19 vaccine intention. These attributes tend to correlate with socioeconomic status; thus it is possible that skilled Myanmar migrants working in professional services might have different levels and domains of vaccination concerns compared to our participants. Future studies should consider expanding the scope of the study to include migrants in higher socioeconomic status.” |
| REVIEWER’s COMMENT  11. Regarding barriers like documentation and logistical challenges, I recommend expanding on potential solutions or policy adjustments that could mitigate these issues. Consider including suggestions such as simplified documentation requirements for vaccination, improved health infrastructure for migrant workers, or incentives for employers to support vaccine uptake. | RESPONSE:  We thank the reviewer for the suggestion. We have added possible policy solutions related to documentation and logistical constraints in the DISCUSSION section as follows:  “This highlights the critical need for more equitable distribution systems that ensure vaccine access regardless of insurance status. To improve vaccine access for migrant populations, supply chain management should be optimized through stratified budgeting, buffer stock systems, and mobile vaccination units, while strengthening cold chain infrastructure.” (Line 420-424)  “Therefore, partnerships with civil society organizations and employers, alongside policy adjustments that include amnesty provisions for undocumented migrants and removal of deterrent documentation requirements, ultimately creating an equitable and efficient vaccination system for all migrants regardless of their status. Moreover, structural barriers should be addressed by simplifying registration processes, removing insurance-based prioritization, and establishing multiple convenient vaccination points with flexible scheduling to accommodate work schedules.” (Line 435-442) |
| REVIEWER’s COMMENT  12. Since documentation status is a critical factor influencing both healthcare access and employment stability, the discussion could benefit from explicitly addressing whether your study participants were documented or undocumented migrant workers. This would provide a more nuanced understanding of the barriers faced, especially regarding vaccine access and adherence to COVID-19 prevention behaviors. | RESPONSE  We thank the reviewer for the comment We allowed both types of migrants. But the migrants that we interviewed were all documented. We realize that such circumstances also limited the generalizability of our findings.  We have included the following remarks as the first limitation in the DISCUSSION section (“Strengths and Limitations” sub-section) as follows (Line paragraph 457-461):  “Firstly, although our protocols did not exclude undocumented migrants, we were only able to recruit documented migrants working in manual labor occupations common in southern Thailand. These circumstances limited the generalizability of our study findings. The barriers among undocumented migrants might have been vastly different from those reported by our participants.” |
| **Conclusion** |  |
| REVIEWER’s COMMENT  13. Line 329-330: It would be helpful to clarify what is meant by ‘both domain’ in the sentence “Supply chain management was a common theme in both domains” | RESPONSE  We thank the reviewer for the comment. We have revised the sentence in the CONCLUSION section as follows (Line 475-477):  “Supply chain management was a common theme in both barriers to vaccination and barriers to adherence to COVID-19 preventive measure.” |
| **Reviewer #2:** |  |
| REVIEWER’s COMMENT  1. The article focuses on research concerning Myanmar migrant workers; however, it lacks clarity regarding the specific group of workers being studied, whether regular or irregular migrants. It is crucial to differentiate between these groups as their experiences vary significantly. For instance, regular migrant workers typically have access to healthcare, whereas irregular migrant workers face restrictions due to their immigration status. | RESPONSE  We thank the reviewer for the comment. We allowed both types of migrants. But the migrants that we interviewed were all documented. We realize that such circumstances also limited the generalizability of our findings. We have tried to revise the manuscript where applicable. |
| REVIEWER’s COMMENT  To address the research gap, a more comprehensive literature review is recommended to enhance the paper. For instance, the article's sample should encompass various groups such as migrant workers, meatworkers, NGOs, and local individuals.  • 'I Doubt Myself and Am Losing Everything I Have since COVID Came'-A Case Study of Mental Health and Coping Strategies among Undocumented Myanmar Migrant Workers in Thailand https://doi.org/10.3390/ijerph192215022 | RESPONSE  We thank the reviewer for the comment. With regard to the research gap, we have revised and included the suggested citation in the INTRODUCTION section as follows (Line 99-109):  “Recent research on COVID-19's impact on vulnerable migrant communities in Thailand incorporated diverse viewpoints from various stakeholders, including representatives of migrant advocacy organizations, NGOs, and community-based support groups [34]. Such data can provide valuable insights for relevant stakeholders that enable planning and preparations for future infection control practices and crises. Building upon this foundation, our qualitative study aims to provide a comprehensive understanding of the barriers to COVID-19 protective behaviors adherence and vaccination among Myanmar migrant workers in Thailand according to the perspectives of three important stakeholders for migrant health: migrant workers, employers, and healthcare providers. This multi-stakeholder approach will generate valuable insights to inform future crisis preparedness and infection control strategies.” |
| REVIEWER’s COMMENT  2. Regarding methodology, the study should provide detailed information on how participants were recruited. Additionally, the low number of migrant workers in the study raises concerns, especially considering that the data collection period occurred after the easing of COVID-19 restrictions in Thailand. The claim that data collection was impeded by COVID-19 restrictions should be verified. | RESPONSE  We thank the reviewer for the comment. We have included the following details in the “Study Participants” sub-section of the METHODS section (Line 145-153):  “Myanmar migrants were identified with the help of the key informants from the Migrant Workers Right Network (MWRN) and the Stella Maris Organization. These key informants were Myanmar Nationalities who had been working in the study area for more than 10 years. They could well acquaint with the Myanmar migrant population within Hat Yai and Pattani and could provide information on places of worksite and residence, types of occupations, legal status, approximate population sizes, and their lifestyles. For Thai employers and Thai healthcare providers, they were also identified by the Thai research coordinator and invited via official request letters, emails, or phone calls and invited to participate in individual in-depth interviews.” |
| REVIEWER’s COMMENT  3. Furthermore, the study should clarify how the data were coded and analyzed, whether through NVivo software or manual coding. | RESPONSE  We thank the reviewer for the comment. We have updated the coding and analysis information in the “Data management and analysis” sub-section of the METHODS section as follows:  “The transcribed data were inductively explored manually, using thematic analysis in accordance with the research objective.”  “The two investigators then separately identified codes, sub-themes, and themes, based on the frequency of occurrence and significance, relevance to research objectives, cultural and contextual sensitivity.” |
| REVIEWER’s COMMENT  Given that the study is purely qualitative, the presentation of findings should follow with qualitative research standards, favoring textual descriptions over tabular formats. | RESPONSE  We thank the reviewer for the comment. We understand and appreciate the reviewer’s advice for the presentation of our findings to follow the standards of qualitative research. However, given the stratification of our participants and the stratification in the presentation of the findings, the authors wish to retain the tabular formats. |
| REVIEWER’s COMMENT  4. Authors are encouraged to devise a more effective chart or diagram to present the findings and integrate direct quotations alongside their interpretations. | RESPONSE  We thank the reviewer for the comment. We have decided to include the coding tree in **Figure 1** and **Figure 2** and added the narrative descriptions to the RESULTS section. Unfortunately, due to space limitations, we could not add direct quotations to the Figures. |
| REVIEWER’S COMMENT  5. The discussion section should incorporate relevant studies from ASEAN countries,  • Preventive knowledge, attitude, and vaccination challenges for COVID-19 among Myanmar refugees and irregular migrants in Malaysia, https://doi.org/10.1016/j.jvacx.2023.100360  • Global Disparities in COVID-19 Vaccine Distribution: A Call for More Integrated Approaches to Address Inequities in Emerging Health Challenges https://doi.org/10.3390/challe14040045 | RESPONSE  We thank the reviewer for the comment. We have added remarks regarding comparison of the findings with those from other ASEAN countries and included the provided citations accordingly in the DISCUSSION section as follows:  “Non-vaccine supply chain management issues included the shortage of essential hygiene items and personal protective equipment (PPE), which could have improved the overall adherence to COVID-19 and other respiratory infection preventive measures [56]. In Malaysia, one study among Myanmar refugees and irregular migrants in Malaysia reported lack of employer support for masks and hand sanitizers during COVID-19 pandemic [57].”  “Vaccine supply chain management barriers in our study included the lack of vaccination opportunity, insufficient vaccine supply, delays in vaccine procurement and delivery, and logistical challenges. Other studies also suggested that logistical issues have driven low COVID-19 vaccine uptake among migrants and racial minorities [60,63]. Disparities in vaccine delivery have been evident, particularly affecting minority and vulnerable communities, such as undocumented migrants in Thailand and the Roma community in Europe [68].” |
| REVIEWER’S COMMENT  6. Additionally, the study should offer practical policy implications, outline its strengths and limitations using sub-heading. | RESPONSE  We thank the reviewer for the comment. We have added appropriate policy implications for each relevant barrier. We also added the strength and limitation sub-heading. |
| REVIEWER’S COMMENT  7. Provide a more detailed and impactful conclusion to enhance its scholarly contribution. | RESPONSE  We thank the reviewer for the comment. We have revised the CONCLUSION section with more relevant policy implications as follows (Line 474-484).  “In this qualitative study, we identified barriers to COVID-19 protective behaviors and vaccination among Myanmar migrants in Thailand. Supply chain management was a common theme in both barriers to vaccination and barriers to adherence to COVID-19 preventive measures. Moreover, each domain also had additional themes, such as lifestyle and habit-related barriers, lack of health education and health promotion, and fear of vaccination. These findings provide potentially useful insights for policymakers and healthcare providers working to enhance vaccine equity among migrant populations and prepare an inclusive plan to respond to future health emergencies. However, limitations regarding the lack of generalizability and social desirability bias should be considered in the interpretation of our study findings. Future research should focus on evaluating targeted interventions that address both systemic barriers and individual concerns, particularly in contexts where migrants face multiple vulnerabilities.” |
