# Supplementary material for: Perspectives of stakeholders on barriers to COVID-19 protective behaviors adherence and vaccination among Myanmar migrant workers in southern Thailand: A qualitative study
Source: PLoS One. 2025 Mar 11;20(3):e0317714. doi: 10.1371/journal.pone.0317714 (PMC11896050; doi:10.1371/journal.pone.0317714)
Supplement: Attachment — Submitted filename: Reviewer comments.pdf [file pone.0317714.s005.pdf]

Thank you very much for your efforts in adding new information on perspectives regarding COVID-19 protective behaviours and vaccination among Myanmar migrant workers, Thai health care providers and Thai employers.

In the attachment, I included my recommendations.

In Introduction session,

Line 65-67: The statement mentions that 1.65 million migrants from Myanmar are in Thailand. It will be better to clarify the year form which this data was sourced.

Please consider adding information about the general health-seeking behaviours of migrants in the study area, especially focusing on irregular migrants. This could provide insights into their specific challenges in accessing COVID-19 preventative measures.

In Methods session,

Line 98-99: It would be beneficial to include information on the non-pharmacological COVID-19 preventative interventions and restrictions in place during the study period, as these had evolved over time.

Line 99-101: It might be useful to explain why these cities were chosen for the study. It would be better to describe the number of migrant workers in those cities. I checked the cited reference, and it is in Thai language only.

Line 116-118: It was mentioned that purposive sampling was used. Providing more detail on how key informants and participants were identified could be more informative.

It will be better to explain how cultural nuances were handled during translation and interviews.

Line 154-161: Using machine-assisted translation tools like Google Translate for initial translations is practical, but it might be worth discussing any limitations or potential inaccuracies associated with this approach or were there any steps taken to ensure the reliability of the translations.

Line 166-172: It might be helpful to provide more detail on the criteria used for identification of themes, sub-themes, and codes. It would be better to specify how VC's (infectious diseases specialist) input was integrated into the final analysis and how any disagreements (if any) between VC and primary investigators were resolved.

In Result session,

Line 185-188: It would be helpful to include any relevant socio-demographic characteristics that might influence the findings or interpretations of the results.
It would be valuable to know whether the migrant workers are documented or undocumented, as this distinction could impact their access to healthcare services and adherence to COVID-19 prevention measures.

In Discussion session,

Line 270-283: It would be beneficial to explore the fear of vaccination in more depth. How does this fear differ across different socioeconomic groups within the migrant worker population? Adding these details would provide more context for your findings and potentially point toward targeted interventions for specific subgroups.

Regarding barriers like documentation and logistical challenges, I recommend expanding on potential solutions or policy adjustments that could mitigate these issues. Consider including suggestions such as simplified documentation requirements for vaccination, improved health infrastructure for migrant workers, or incentives for employers to support vaccine uptake.

Since documentation status is a critical factor influencing both healthcare access and employment stability, the discussion could benefit from explicitly addressing whether your study participants were documented or undocumented migrant workers. This would provide a more nuanced understanding of the barriers faced, especially regarding vaccine access and adherence to COVID-19 prevention behaviors.

In Conclusion session,

Line 329-330: It would be helpful to clarify what is meant by 'both domain' in the sentence "Supply chain management was a common theme in both domains".
